# Efficacy of chlorhexidine, hydrogen peroxide and tulsi extract mouthwash in reducing halitosis using spectrophotometric analysis: A randomized controlled trial

**DOI:** 10.4317/jced.55523

**Published:** 2019-05-01

**Authors:** Kriti Sharma, Shashidhar Acharya, Eshan Verma, Deepak Singhal, Nishu Singla

**Affiliations:** 1 Dept. of Public Health Dentistry, Manipal College of Dental Sciences, Manipal Academy of Higher education, Madhav Nagar, Manipal, Karnataka

## Abstract

**Background:**

To evaluate the efficacy of tulsi extract mouthrinse in reducing halitosis as compared to chlorhexidine and hydrogen peroxide mouthrinses using spectrophotometric analysis.

**Material and Methods:**

It was a parallel, single center, double blinded randomized controlled trial of 15 days duration. A total of 300 participants were screened, out of which 45 subjects those fulfilled inclusion criteria of age range 17-35 years were included in the trial. The participants were randomly provided with tulsi, hydrogen peroxide or chlorhexidine mouthwashes (control group) to use 10 ml twice daily for 15 days. Clinical measurements were taken at baseline and 15 days using arbitrary 0-5 scale for organoleptic odor assessments, spectrophotometric analysis for Volatile Sulphide Compounds (VSCs) from saliva samples, Löe & Silness gingival index and Silness & Löe plaque index.

**Results:**

After intervention, organoleptic, VSCs, gingival and plaque scores showed statistically significant decrease in all the three study groups. The mean percentage reduction in VSC and organoleptic scores was significantly greater in chlorhexidine and hydrogen peroxide groups than in the tulsi group and the mean percentage reduction in plaque and gingival scores was significantly greater in chlorhexidine group than in hydrogen peroxide and tulsi group.

**Conclusions:**

Tulsi may not have the efficacy of chlorhexidine and hydrogen peroxide mouthrinses. But, Tulsi is effective in reducing halitosis, plaque and gingivitis and with its lack of side effects & cost effectiveness, can be an effective & economic tool to deal with halitosis. The spectrophotometric technique appears to be a promising method for evaluation of oral malodor.

** Key words:**Halitosis, tulsi mouthwash, chlorhexidine, hydrogen peroxide, spectrophotometric analysis.

## Introduction

Halitosis is one of the society’s oldest and most troublesome social maladies. It has been recorded in literature since 1500 B.C. It is commonly described as bad breath, unpleasant or nasty smell coming from the mouth. It is a common and universal affliction suffered by many people irrespective of age, sex, social status. Over half of the population experience it but only one out of ten adults suffers from its severity that requires medical help (1,2). Halitosis has become a major health concern among the general public because it causes significant amount of social disharmony, embarrassment, frustration, despair and often leads toward social and professional isolation and marital problems (3). It is expected to be the third most common cause for obtaining dental treatment, after dental caries and gum problems (4,5).

A number of reasons both intra oral or extra oral have been identified for halitosis such as tongue coating, gingivitis, periodontitis, sinusitis, post-nasal drip, diabetes mellitus, hepatic cirrhosis, uremia, internal bleeding or lung carcinoma (6,7). Bad breath which is persistent or chronic may indicate towards a more serious condition. However, in most of the cases bad breath originates in the mouth itself (8,9). It is usually due to the proteins degradation by some particular bacteria releasing volatile sulphur compounds (VSCs) (9). Dimethyl sulphide, hydrogen sulphide and methyl mercaptan are the major constituents in these volatile sulphur compounds which can be found in saliva or gingival crevicular (9,10).

Chlorhexidine is one of the most popularly tested antimicrobial agent for its efficacy in the treatment of oral bad breath (11). It has been shown to be successful in reducing the bacterial load and thus is seen as potentially effective agent in controlling halitosis, but it has disadvantages of increased tooth staining (10). Bad breath may be temporarily reduced by using a hydrogen peroxide rinse. Hydrogen peroxide at a concentration of 1.5% can be taken as an oral antiseptic. Hydrogen peroxide is a powerful oxidizer which kills most bacteria, including useful aerobic bacteria. A study reported that hydrogen peroxide rinses are associated with mucosal abnormalities and hence, usually not advised for oral care (12). To avoid these side effects, alternatives of ayurvedic formulations to Chlorhexidine and hydrogen peroxide can be used. Tulsi (Ocimum sanctum) is a small plant, sub-shrub which has multiple uses. Ayurveda mentions the importance of medicinal uses of it (13). Chewing few tulsi leaves are known to be quite effective for curing ulcers and infections in the oral cavity. The powdered of sun dried tulsi leaves can be used for brushing teeth. It is also good for curing bad breath, pyorrhea and other gum disorders (14).

Various methods known for halitosis detection are broadly classified as direct methods (Organoleptic method, Gas Chromatography, Sulphide Monitoring - Halimeter, Breath Alert) and indirect methods (BANA test, Bacterial Culture, Direct Bacterial Smears, Enzyme Assay) (11,15). A lot of information on the crucial aspects involved in halitosis formation has been generated from studies done on incubated whole saliva received from humans (16). In this study a newer technique, “spectrophotometric analysis from headspace of suspended saliva” was attempted to assess the reduction in mercaptans levels following the use of chlorhexidine, hydrogen peroxide and tulsi mouthrinse (17). So, the aim of the present study was to determine the effectiveness of tulsi extract mouthrinse, in reducing halitosis as compared to Chlorhexidine and hydrogen peroxide mouthrinses, using spectrophotometric analysis.

## Material and Methods

It was a parallel design, single center, double blinded randomized controlled trial of 15 days duration. The ethical approval to conduct the study was taken from the Ethics Committee of the University. The sample was selected among patients from dental outreach center, TMA Pai Hospital, Udupi. The participants were informed regarding the study and written consent was obtained for participation in the study. The eligibility criteria which had been used for inclusion of the subjects in the study were those subjects presented halitosis of oral origin and those subjects with an organoleptic score > 1 by means of an arbitrary 0-5 scale (ranged from no malodor to offensive malodor) (18). The following subjects were excluded those who were not willing to participate, subjects those were smokers, subjects undergoing antibiotic or other antimicrobial therapy, subjects with any medically compromised conditions contraindicating the oral examination and subjects with active periodontitis and multiple carious lesions.

Sample Size of 45 subjects was calculated taking a significance level (α) of 0.05, power of study (β) of 80% and minimum expected difference between the two means of 0.1. (19) Assuming possible losses of 20%, the numbers was adjusted to 15 subjects per group. A total of 300 participants were screened, out of which 45 subjects, who agreed to participate and fulfilled the inclusion criteria were included in the study. The criteria for screening was the presence of halitosis after patients were instructed to refrain from tooth brushing, mouth rinsing, eating or drinking for at least 2 hours prior to the clinical measurements and to rinse with water at least 20 minutes before the measurement to protect dry mouth effect and not to talk for at least five minutes.

The selected 45 subjects were randomly distributed into three groups by a lottery method. Following the baseline examination and saliva collection, the participants in three groups were provided with either of the mouthrinse by an assistant, who was not the part of the study. The control group was provided with the Chlorhexidine mouthwash (commercially available as 0.2 % w/v Hexidine mouthwash) and other study groups were provided with Hydrogen Peroxide mouthwash (commercially available as 1.5% w/v Speedrox) and Tulsi mouthwash (Prepared at 4% from Tulsi extract bought from Nirogam Pvt Lmt). The mouthrinses were provided in opaque containers to Group 1: Chlorhexidine mouthwash, Group 2: Hydrogen Peroxide mouthwash and Group 3: Tulsi mouthwash, which were coded by the assistant. The investigator was blinded for the mouthwash that was being given to the patients. The patients were also initially blinded at the time of allocation of the mouthrinses to them. They were advised to use 10 ml twice daily for 15 days irrespective of their oral hygiene practices.

Evaluation of participants’ compliance was done after the first 7 days; a reminder call was made by the investigator to enquire about the compliance of the instructions. They were told to use the mouthwash twice daily for the next 7 days. At the 15th day, they were told to return the remaining mouthwash and the content was measured. At the end of the experiment, oral hygiene instructions and oral prophylaxis were given to all the participants. Single trained and calibrated investigator collected all the clinical data at baseline and 15 days using arbitrary 0-5 scale for organoleptic odor assessments for which subjects were asked to not to open their mouth for 1 min and then to exhale air slowly at approx. 10 cm distance away from the nose of the investigator. Gingivitis was evaluated using Löe and Silness index (1963) (20) and plaque with Silness and Löe index (1964) (21) and at last 5ml of the subjects’ unstimulated saliva was collected in 10ml glass vials which were then sealed using rubber septum and incubated at 37°C for 24 hours.

A new method was used to measure the Volatile Sulphide Compounds (VSCs) from the headspace of suspended saliva samples. In this method, mercaptans and other sulfur containing compounds which gets collected over the saliva were then aspirated using syringes half filled with aqueous solution of mercuric acetate-acetic acid. Then the aspirated air present in the needle was passed through a strongly acid solution of N,N -dimethyl-p-phenylenediamine and ferric chloride. The collected mercaptans were subsequently determined by spectrophotometric measurement of the red complex formed by the reaction between mercaptans and a strongly acid solution of N, N-dimethyl-p-phenylenediamine and ferric chloride. These measurements were made using a UV spectrophotometer at 550 nm. This technique is used for detection of the presence of mercaptans in the atmosphere. It detected the reduction in the level of the mercaptans after intervention with different mouth rinses.

Data of the study was assessed using the Statistical Package for Social Sciences (SPSS) version 20.0. Comparison of various scores among three study groups at baseline was done using ANOVA test. Comparison of various scores at baseline and post intervention was done using paired t test. Percentage reduction was computed in SPSS by {(pre – post)÷pre} ×100 for various scores and mean percentage reduction among three groups were compared with ANOVA and post hoc Tukey test. Correlation between the results of the organoleptic test and the spectrophotometric values was evaluated using the Spearman correlation coefficient. A *p*-value ≤ 0.05 was considered to be statistically significant.

## Results

The present study constituted sample size of 45 subjects, 15 each in Chlorhexidine, Hydrogen peroxide and Tulsi groups. The mean age of study subjects was 24.78 years (SD 4.89, Range 17-35). There was no loss to follow up in the study and on evaluation of participants’ compliance, it was found that the remaining content of the mouthwash was not different at day 15 in all the three groups and thus indirectly indicated good participants’ compliance. Comparison between the three study groups showed no significant difference at the baseline in terms of means of VSCs’ scores, organoleptic scores, plaque scores and gingivitis scores ( Table 1).  Table 2 shows pre and post intervention comparison of VSC scores, organoleptic assessment scores, and plaque and gingivitis scores at the baseline and at 15 days of intervention in three study groups. It was found that after intervention VSCs, organoleptic scores, plaque and gingival scores showed statistically significant decrease in all the three groups.  Table 3 shows the comparison of the mean percentage reduction in VSC scores, organoleptic scores, plaque and gingivitis scores among three study groups after 15 days of follow-up. It was found that the change in VSC and organoleptic scores was significantly greater in chlorhexidine and hydrogen peroxide groups than in the tulsi group. Similarly, the change in plaque and gingival scores was significantly greater in chlorhexidine group than in hydrogen peroxide and tulsi group.

**Table 1:**
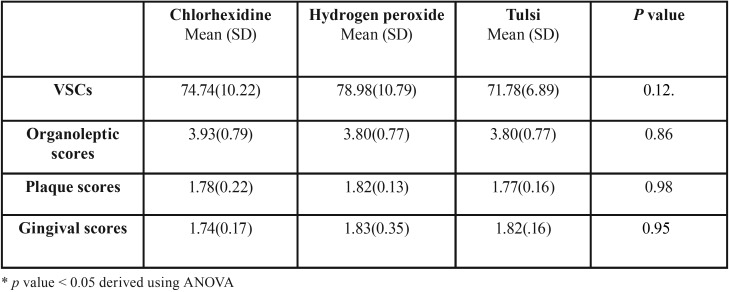
Comparison of volatile sulphide compound scores (VSC), organoleptic scores, plaque and gingival scores at baseline among three study groups.

**Table 2:**
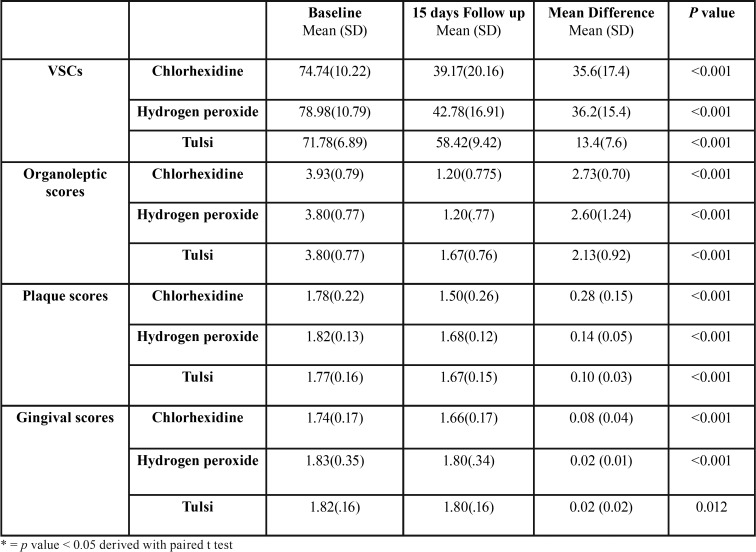
Comparison of volatile sulphide compound scores (VSC), organoleptic scores, plaque and gingival scores at baseline and 15 days post intervention among three study groups.

**Table 3:**
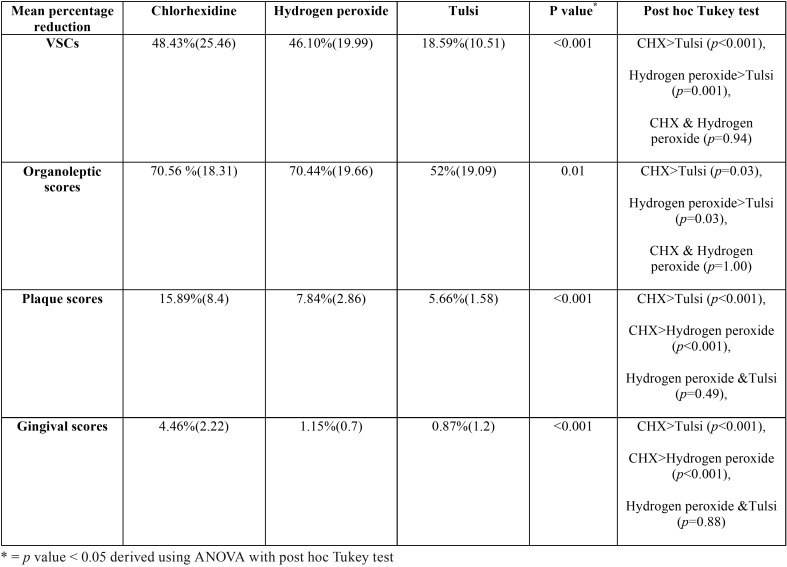
Mean percentage reduction in volatile sulphide compound scores (VSC), organoleptic scores, plaque and gingival scores among three study groups.

**Figure 1:**
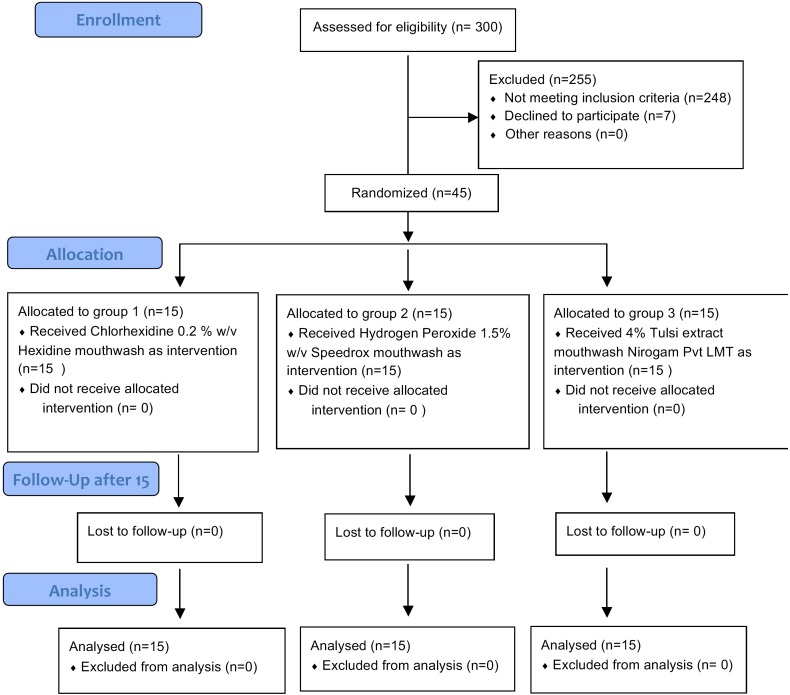
CONSORT flow diagram.

In addition, organoleptic scores were positively correlated with the scores of VSCs and the Spearman’s correlation coefficient was found to be 0.504, (Fig. 1).

## Discussion

The present trial was done to evaluate the effectiveness of Chlorhexidine, Hydrogen peroxide and Tulsi extract mouthrinses in reducing halitosis of oral origin. Participants with moderate to severe halitosis were included in the trial, as such participant’s baseline VSC levels will be high enough to evaluate the effects of formulations. Spectrophotometric analysis was used to detect VSCs because of their superior reproducibility and sensitivity, when compared with organoleptic measurements. Chlorhexidine di-gluconate is the most extensively studied and is considered gold standard for anti plaque and anti gingivitis agent. The most commonly prescribed concentration is 0.2% hence, this was considered in the study (22). In the present study, chlorhexidine significantly reduced the mean VSC, organoleptic, gingival and plaque scores from baseline to day 15. The potential of hydrogen peroxide to reduce oral malodor was investigated in-vitro and in-vivo studies (23). Hydrogen peroxide is a powerful oxidizer which kills most bacteria, including useful aerobic bacteria (12). However, one study has reported that higher concentrated hydrogen peroxide rinses they might cause some mucosal abnormalities (13). In the present study, 1.5% concentration was considered. The results showed that there was a statistically significant difference in the mean VSC, organoleptic, plaque and gingival scores from baseline to day 15.

The pharmacological activity of Ocimum sanctum (Tulsi) has been well documented in the field of dentistry. In this study, tulsi mouthrinse was formulated and its effect on malodor was assessed after 15 days. A study had analyzed the effect of various concentrations of the Ocimum sanctum extract ranging from 0.5 to 10%, and it was observed that a 4% concentration of the extract was optimum as an antibacterial agent against bacterial flora of the oral cavity; thus, in the present study, a concentration of 4% was used (24). Ocimum sanctum has been tested against a variety of microorganisms like Candida albicans, Staphyloccus aureus, enteric pathogens, Klebsiella, Escherichia coli and Proteus (25). In the present study, the Ocimum sanctum extract mouthwash prevented plaque formation during the trial. Possible explanation for this effect is the antibacterial agents present in Ocimum sanctum i.e. Eugenol (l-hydroxy-2-methoxy-4-allylbenzene) Ursolic acid (2,3,4,5,6,6a, 7,8,8a, 10,11,12,13 14-btetradecahydro-1H-picene-4a-carboxylic acid) and Carvacrol (5-isopropyl-2-methylphenol), Linalool (3,7-dimethylocta-1,6-dien-3-ol), Limatrol, Caryophyllene (4,11,11-trimethyl-8-methylene-bicyclo [7.2.0]undec-4-ene), Methyl carvicol (also called Estragol: 1- allyl-4-methoxybenzene). In addition, the stem and leaves of Ocimum sanctum contain a variety of constituents that may have antibacterial activity, including saponins, flavonoids, triterpenoids and tannins that forms high molecular weight complexes with soluble proteins in saliva, increases bacterial lysis on the tooth surface and saliva (26,27). This study showed a significant reduction in VSCs, organoleptic, plaque and gingival scores in tulsi extract mouthwash group, which can be attributed to compounds isolated from tulsi extract. However, after 15 days of intervention, the mean percentage reduction in VSC and organoleptic scores in chlorhexidine and hydrogen peroxide groups was significantly greater than in the tulsi mouthrinse group. Also, the mean percentage reduction in plaque and gingival scores in chlorhexidine group was significantly greater than in hydrogen peroxide and tulsi mouthrinse groups. Hence, it can be concluded that the tulsi mouthrinse, though effective in reducing VSCs which are responsible for halitosis, does not have the efficacy of chlorhexidine and hydrogen peroxide mouthrinses.

Considering the fact that the mouth rinses available presently in the market are chemically based, costly and have side effects, which restrict their use, a cost effective and easily available herbs as adjuvant to oral hygiene maintenance may have a far reaching effects on the prevention and prevalence of oral diseases. Comparison with the other studies could not be carried out as, tulsi extract mouthrinse has not been studied separately in reducing halitosis. In combination with other herbal mouth rinses, the anti-plaque and anti-gingivitis effect has been seen (28) another study reported that tulsi mouthrinse as an effective mouthwash owing to its ability in decreasing periodontal indices by reducing plaque accumulation, gingival inflammation and bleeding. It has no side effect as compared to chlorhexidine (29).

To eliminate any bias, the participants were asked to follow their routine oral hygiene practices. Examination by the dentist might have introduced some bias in the study, as the participants might get over cautious and start following meticulous oral hygiene practices. But such a phenomenon might have occurred in all the groups. Results of the study favors promotion of Ocimum sanctum as a mouth rinse among rural communities especially belonging to low socioeconomic strata as Ocimum sanctum is easily accessible and is cheap and safe alternative to chlorhexidine. However as this is the first attempt to assess the effect of Ocimum sanctum on halitosis, clinical trial of longer duration with a larger sample size should play a vital role in commercialization of Ocimum sanctum mouthwash.

## Conclusions

It can be concluded that tulsi is effective in reducing halitosis, plaque and gingivitis. It may not have the efficacy of chlorhexidine and hydrogen peroxide mouthrinses. But Tulsi, with its lack of side effects & cost effectiveness, can be an effective & economic tool to deal with halitosis. In a public health setting, where regular follow-ups are difficult due to lack of compliance on part of the patient, Tulsi can be safely prescribed to such patients for longer duration of time. The spectrophotometric technique employed in this study appears to be a promising new method for evaluation of oral malodor.
